# Development and Validation of an Affinity Chromatography-Protein G Method for IgG Quantification

**DOI:** 10.1155/2014/487101

**Published:** 2014-10-27

**Authors:** Lesly Paradina Fernández, Loany Calvo, Lisel Viña

**Affiliations:** ^1^Department of Quality Control, Center of Molecular Immunology, 11600 Havana, Cuba; ^2^Biochemistry Laboratory, Quality Control Department, Center of Molecular Immunology, 11600 Havana, Cuba

## Abstract

Nimotuzumab, an IgG that recognizes the epidermal growth factor receptor (EGF-R) overexpressed in some tumors, is used in the treatment of advanced head and neck cancer. For the quantification of this protein in cell culture supernatants, protein G-HPLC affinity chromatography is used due to its high affinity and specificity for antibodies of this class. The technique relies on the comparison of the area under the curve of the elution peak of the samples to be evaluated versus to a calibration curve of well-known concentrations and was validated by assessment of its robustness, specificity, repeatability, intermediate precision, accuracy, linearity, limit of detection, limit of quantification, and range. According to results of the study all validation parameters fulfilled the preestablished acceptance criteria and demonstrated the feasibility of the assay for the analysis of samples of cell culture supernatant as well as drug product.

## 1. Introduction

Therapeutic use is of the most important applications of monoclonal antibodies (Mabs). The recent development of engineered humanized monoclonal antibodies has increased their therapeutic efficacy and decreased their toxicity, expanding their potential for the treatment of cancer [1].

Tumors of epithelial origin, among which we have head and neck cancers, are one of the leading causes of death worldwide. Nimotuzumab is used to treat these entities. This is a humanized monoclonal antibody (mAb) expressed in NS0 cells and obtained at the Center of Molecular Immunology by genetic engineering techniques [2, 3] by the fusion of the hypervariable regions (CDR) of murine origin with the variable region frameworks and the constant regions of the heavy and light chains of human origin and back mutation of critical residues. This antibody recognizes the epidermal growth factor receptor (EGFR) that is overexpressed in epithelial tumors and is associated with malignant transformation process [4, 5].

Nimotuzumab, being a human IgG class 1 (hIgG1) molecule, is composed of two identical heavy chains (HC ~ 50 kDa) and two identical light chains (LC ~ 25 kDa) [6]. This glycoprotein presents one N-glycosylation site in each heavy chain mainly comprising nonsialylated biantennary fucosylated structures [2, 7]. The presence of oligosaccharides is critical for the structure, stability, and biological function of the antibody [8].

Nimotuzumab has been extensively and rigorously characterized as requested for all recombinant proteins intended for use in human therapy [2, 9].* In vitro* and* in vivo* studies have demonstrated potent antitumor activity and antiangiogenic and proapoptotic so this antibody plays an important role as a therapeutic agent [10, 11] as demonstrated by the results of the several clinical studies in which this molecule has been evaluated [12].

As for any therapeutic product, a tight control is needed to monitor the production and quality of the final product [13].

The quantification from cells supernatant is required for the control of the purification of this recombinant protein. For this reason the implementation of a selective method, capable of determining the amount of IgG in cells supernatant, is necessary.

Several methods can be used for the specific quantification of antibodies on complex samples (like culture supernatant). Ideally, the method should be fast and simple and provide high throughput. It should also provide an adequate level of specificity and sensitivity due to the presence of impurities and its low concentration. ELISA is a method that fulfills all these criteria but can be labor intensive and can be more affected by matrix components than interfering with the antigen-antibody reaction. HPLC represents an alternative method in those cases. For antibody quantitation, reverse phase and affinity based methods (using protein A or G) have been used [14, 15]. Reverse phase has the advantage of using cheaper columns and common solvents. But for companies handling several different antibodies, it might be difficult to find a common procedure suitable for all of them. This problem is overcome by the use of affinity columns, as long as all the products belong to a suitable IgG isotype.

In this sense the determination by affinity chromatography using protein G by HPLC is an attractive method because it has very high affinity and specificity for the human IgG antibodies [16]. On the other hand, this technique has several advantages over other conventional methods because it provides a high capacity and selectivity [17] and allows the removal of specific contaminants from biological samples [18].

Regulatory agencies require that this technique, like all those used for the monitoring of therapeutic biotechnology products, must be validated to confirm that the analytical method used for a specific test is suitable for the proposed use, ensuring its reliability [19].

The validation of a specific method must be carried out using laboratory experiments where the samples or standards used are similar to the samples routinely analyzed. The parameters studied during validation of an analytical method must be defined in advance as described in the International Conference on Harmonization (ICH) [20].

This study is vital if one considers that the use of a nonvalidated technique for the control of some critical parameter may risk the patient's life due to the use of products that do not have sufficient safety and/or efficacy.

## 2. Experimental

### 2.1. Description of the Samples

The drug product of Nimotuzumab used for the standard preparations was contained in a formulation buffer composed by phosphate buffer, sodium chloride, and polysorbate 80, pH 6.5–7.5.

The culture medium of the supernatant was PFHM-II (protein-free additives) pluronic C, antifoaming, NS0 cell line components, and Nimotuzumab.

### 2.2. Preparation of Calibration Curve

The calibration curve was prepared using a reference material of Nimotuzumab (MRT) (Havana, Cuba) diluted in mobile phase A (2, 4, 6, 8, and 10 *μ*g).

### 2.3. Analytical Procedure

In all cases the area of the elution peak was determined and plotted against the IgG concentration of the standards to construct the calibration curve (linear regression). The result of the supernatant samples was obtained by interpolation of the area of the elution peak into the calibration curve.

### 2.4. Instrumentation and Chromatographic Conditions

The affinity chromatography was performed in a high performance liquid chromatography (HPLC) (Shimadzu, Japan) system consisting of a quaternary pump, a solvent degasser, a column oven, and a variable-wavelength UV detector. Operation parameters were fixed and controlled through a personal computer using LC Solution software version 1.25SP1.

A POROS G/20 of 100 mm × 4.6 mm (Applied Biosystems, California) column was employed [21] using three mobile phases (A: 0.05 M phosphate buffer, 0.02% sodium azide, pH 7.5 for equilibration of the column; B: 0.25 M glycine, pH 2.5 for product elution; C: 0.25 M glycine, pH 6.1) for washing of impurities before product elution. The flow rate of the mobile phase was 2 mL/min and the column temperature was 25°C. The injection volume of the samples and curve calibration were 50 *μ*L. The final profile is obtained after subtraction of the profile of a blank to assist in the integration of the baseline.

### 2.5. Optimization of Affinity Chromatography Method

The mobile phases B and C were modified varying the molarity values to 0.05 M, 0.1 M, 0.25 M, and 0.5 M and the tailing factor of the elution peak from each chromatogram was calculated.

### 2.6. Validation of Method

#### 2.6.1. Robustness

To determine the robustness of the analytical method 7 factors were evaluated (pH of mobile phases A and B; molarity of mobile phases A, B, and C; column batch; oven temperature) using a Plackett-Burman factorial experimental design. The variable used for statistical treatment of the data was the IgG concentration from Nimotuzumab reference material, and the effect of each factor on the response was calculated as defined by Van der Hayden et al. [22–25]. Acceptance criteria are as follows: the effects of the analyze factors should not be more than the margin of error determined by the method.

#### 2.6.2. Specificity

The interference of the matrix was evaluated from a run using formulation buffer, cell culture supernatant from nontransfected NS0 cells (SN NS0), and mobile phase A as independent samples. Besides, the parallelism of the standard curves prepared in mobile phase A (MPA) was tested with respect to curves prepared in culture media: culture medium PFHM II (CM PFHMII) and culture medium PFCHO (CM PFCHO). The parallelism test was performed using a regression analysis comparing the slopes and intercept between calibration curves. Acceptance criteria are as follows: there should be no signals on the profiles from samples not containing the analyte during the elution time of the product. Additionally, the curves prepared on different media should have similar slopes and intercept.

#### 2.6.3. Precision

Repeatability and intermediate precision studies were assessed from the analysis of four different batches of cell culture supernatants (SN) during two experimental days using two different equipments. The statistical analysis was performed by analysis of variance (ANOVA) [26, 27] and the %RSD of repeatability and intermediate precision was determined from the standard deviation of the method and the analyzed factors (day/equipment). Acceptance criteria are as follows: the %RSD for the repeatability and intermediate precision analysis should not be more than 2% and 3%, respectively.

#### 2.6.4. Linearity

The linearity of the method was evaluatedinjecting different amounts of IgG (2, 4, 6, 8, and 10 *μ*g). A curve was plotted between the area of the elution peak versus the applied IgG mass. The determination coefficient and slope were calculated by linear regression analysis. Acceptance criteria are as follows: the determination coefficient should not be less than 0.98, and the slope should be different from zero.

#### 2.6.5. Accuracy

The IgG concentration of different batches of drug product and reference material of Nimotuzumab (MR) was determined by HPLC-protein G and DO_280 nm_ (used as a reference method). The ratio between the concentrations for both test methods (IgG concentration HPLC/IgG concentration DO_280 nm_) was calculated. In addition different concentrations of reference material of Nimotuzumab were evaluated in duplicate, comparing the observed and theoretical values. Acceptance criteria are as follows: % recovery should be 80–120%.

#### 2.6.6. Limit of Detection (LOD) and Limit of Quantification (LOQ)

The detection and quantification limit of the test method was calculated from the standard deviation and the slope of the calibration curve used in the linearity study as described in ICH guidelines [19]. Acceptance criteria are as follows: the LOQ should not be more than 2 *μ*g.

#### 2.6.7. Range

The range was established from the results obtained during precision studies, linearity and accuracy.

## 3. Results and Discussion

### 3.1. Optimization of Affinity Chromatography Method

**Figure 1:**
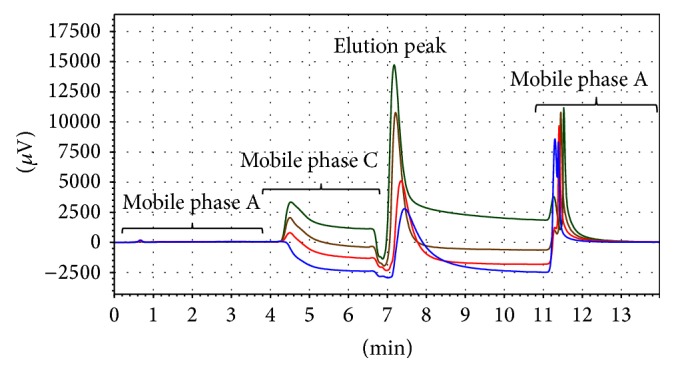
Chromatographic profile for IgG (6 *μ*g) obtained by varying the molarity of mobile phases B and C (0.05 M, 0.1 M, 0.25 M, and 0.5 M).

The original method used 0.05 M glycine, pH 6.1 and 0.05 M glycine, pH 2.5 as mobile phases for washing, and elution, respectively. In these conditions the peak had a broad shape with a long tail. By increasing the molarity of the above-mentioned solutions to 0.25 and 0.5 M, it was possible to reduce the tailing factor below 2.0, as recommended by USP [28, 29] (see Table 1 and Figure 1). The use of 0.25 M glycine solutions allowed a better analysis of the chromatograms since peak start and end points are easier to assign. The final chromatographic conditions are those described in Section 2.4.

### 3.2. Robustness

**Figure 2:**
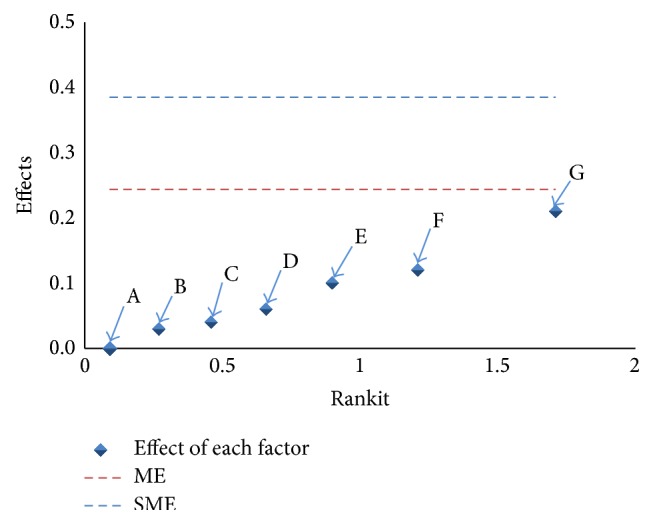
Effects of different factors on the IgG concentration, where A: molarity of mobile phase A, B: molarity of mobile phase B, C: pH of mobile phase A, D: batch of column, E: molarity of mobile phase C, F: pH of mobile phase B, and G: oven temperature.

Table 2 shows the Plackett-Burman factorial experimental design and the IgG concentration from Nimotuzumab reference material used for the evaluation of the robustness study. The evaluation of the significance of the effects obtained by each parameter on the IgG concentration is shown in Figure 2. The effects represent differences in the result of IgG concentration obtained for upper and lower values of each factor. Subsequently the margin of error (ME) and simultaneous margin of error (SME) were calculated. These margins represent the limit where the effects begin to be considered as significant [25].

Figure 2 shows that all the effects caused by the factors studied are below the ME and SME limits, so the IgG concentration determined is not significantly affected by any of these factors. The effect of oven temperature is close to the ME limit. Although it does not constitute a significant factor because it does not exceed the limit, a tight control must be established over the levels of variation for this parameter compared to the rest.

This study demonstrates the robustness of this analytical procedure as a measure of its capacity to remain unaffected by small but deliberate variations in method parameters and provides an indication of its reliability during normal usage [20, 25].

### 3.3. Specificity

**Figure 3:**
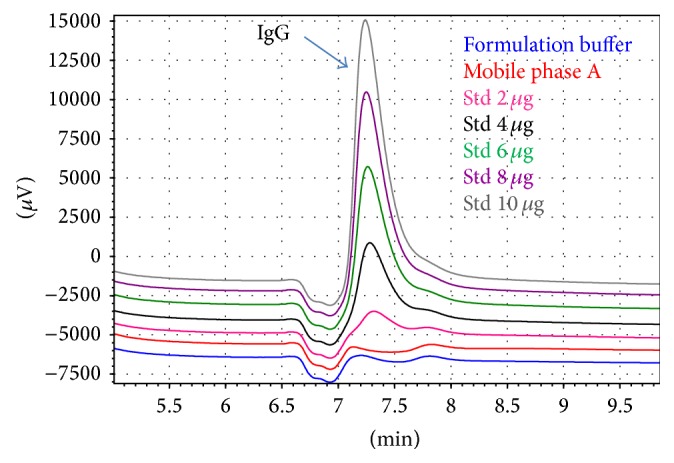
Section of chromatographic profile obtained in the specificity study where the IgG elution peak for different types of samples can be observed.

Since Nimotuzumab is obtained from transfected NS0 cells and fermented in protein-free medium, in the process another source of antibodies does not exist that may interfere with the response of the method. The chromatograms of Figure 3 show the absence of ghost peaks in the formulation buffer. This is expected because in the buffer there should not be substances generating signals, and also in the absence of analyte the peaks must not be detected. This similar behavior was expected between the mobile phase A and the formulation buffer because in both cases the composition is similar. Both solutions have phosphate salts. The formulation buffer has other components (sodium chloride and polysorbate 80) which must not emit signal on the test. These results support the use of the mobile phase A into preparation of calibration curve.

**Figure 4:**
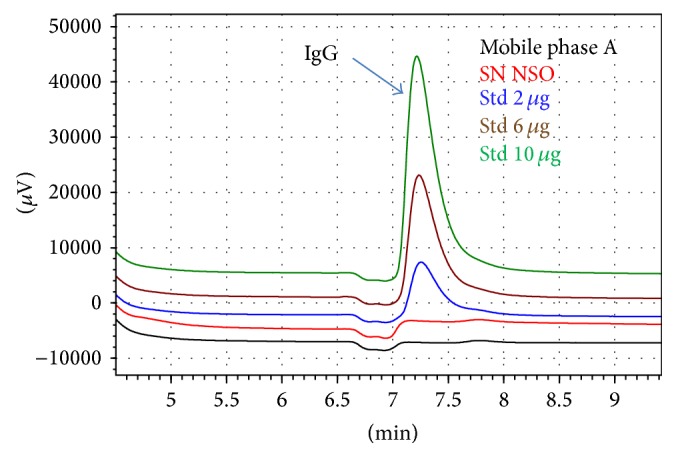
Section of chromatographic profile obtained in the specificity study, where IgG elution peak for different types of samples can be observed.

Additionally, a similar behavior was observed between the SN NS0 and mobile phase A (Figure 4). This result is again expected because this supernatant was obtained from a nontransfected NS0 cell line and therefore did not produce the antibody, confirming then that other host cell related proteins do not interfere with the results.

The parallelism test was performed using a regression analysis comparing the slopes and intercepts between the calibration curves prepared in different matrixes. In all cases, the parallelism was demonstrated between the calibration curves and obtained high correlation coefficient values (Table 3).

The statistical analysis demonstrated that the slopes were significantly different from 0 (*P* < 0.05); thus there is a correlation between the peak area and the IgG mass. Furthermore significant differences were not observed between the slopes and intercepts of the calibration curves prepared in culture medium, with respect to the calibration curve prepared in mobile phase A (*P* > 0.05) which is indicative not only of parallelism but also of coincidence between them. Therefore the specificity study reveals that there are no interference contributions from the components of the different culture medium used, which justifies the feasibility of using this method for the analysis of samples in CM PFCHO or CM PFHMII culture medium. All of the above demonstrates the specificity of the method to unequivocally evaluate the analyte [19].

### 3.4. Precision

In the repeatability study the %RSD was less than 1%, demonstrating the repeatability of the method between independent determinations using the same operating conditions in a small time interval [26, 30]. The factors day and equipment did not contribute significantly to the overall variability of the results in our working conditions. Therefore intermediate precision results are similar to those of repeatability, complying the acceptance criteria (%RSD less than 3%).

It can be concluded that the method for quantification of IgG by protein G is precise.

### 3.5. Linearity

**Figure 5:**
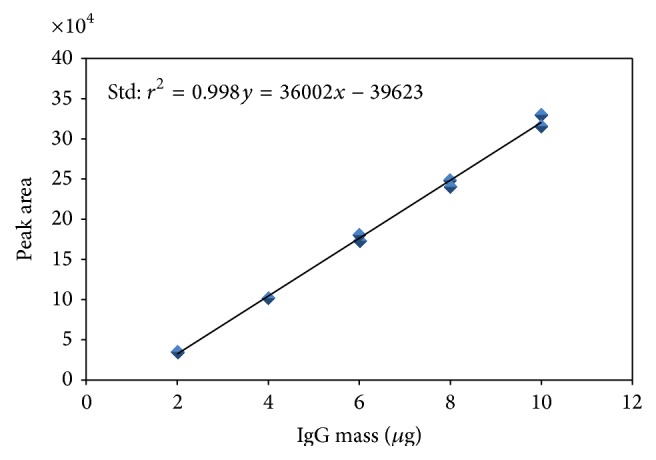
Relationship between the elution peak area versus IgG mass.

The linearity study was performed plotting the area of the elution peak versus the applied IgG mass (Figure 5). The determination coefficient (*r* ^2^) was greater than 0.98, which ensures that a high correlation exists between the variables peak area and applied antibody mass. Moreover, the statistical regression analysis showed a *P* < 0.05 (*P* = 0.000) for the slope being significantly different from 0 and the stadigraph lack of fit showed a *P* > 0.05 (*P* = 0.628), demonstrating the linearity of the method from its ability to obtain results directly proportional to the concentration of analyte in the sample within the given range [20].

### 3.6. Accuracy

**Figure 6:**
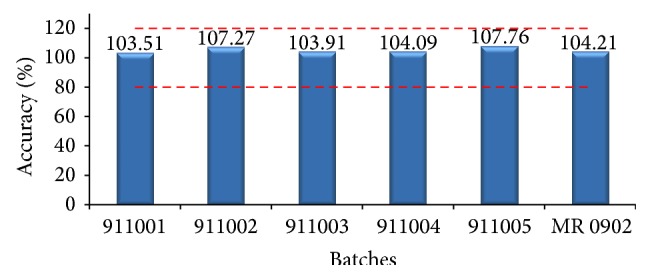
Relationship between IgG concentration HPLC/IgG concentration DO_280 nm_ for different batches of drug product where the dashed lines represent the acceptance criteria of validation.

The study of accuracy allows expressing the closeness of a value that is accepted as standard or an accepted reference value and the value obtained [31]. The relationship between the concentrations for both test methods (IgG concentration HPLC / IgG concentration DO_280 nm_) was calculated (Figure 6). The results varied within 10% for all batches evaluated, complying the acceptance criteria established (±20%) in this validation.

Table 4 shows the comparison of the observed values with respect to the theoretical ones. A very high percent of recovery was obtained demonstrating the method accuracy in the range of 2–10 *μ*g.

### 3.7. Limit of Detection and Quantification

The detection and quantitation limit yielded similar results (1.1 *μ*g) because the slope of the calibration curve causes that the differences between the corresponding values of IgG mass are negligible. The above-mentioned mass corresponds to a product concentration of 0.022 mg/mL.

### 3.8. Range

According to the results obtained during precision, linearity, and accuracy studies, the range of validity of the test is established between 2 and 10 *μ*g of antibody applied to the column.

## 4. Conclusions

The method developed for the quantification of IgG for affinity chromatography using protein G is robust, specific, precise, linear, and accurate. Therefore this method can be used for the analysis of culture supernatant and drug product and could be widely used for the routine analysis and quality control because is an attractive, simple selective assay.

## Figures and Tables

**Table 1:** Comparison between the tailing factor of IgG elution peak for different molarity values in the mobile phases B and C.

Mobile phases B and C molarity (M)	Tailing factor (%)	Specification USP tailing
0.05	2.846	≤2%
0.1	2.581	
0.25	1.941	
0.5	1.794	

**Table 2:** Factors, levels, design, and experimental results (IgG conc) obtained during the Plackett-Burman factorial study for robustness.

Exp	pH mobile phase A	Molarity mobile phase A (M)	pH mobile phase B	Molarity mobile phase B (M)	Batch of column	Molarity mobile phase C (M)	Oven temperature (°C)	IgG conc. (mg/mL)
1	7,6	0,051	2,5	0,24	1^a^	0,24	23	5,37
2	7,4	0,051	2,5	0,26	2^b^	0,26	23	5,43
3	7,4	0,049	2,5	0,26	1^a^	0,24	27	5,59
4	7,6	0,049	2,3	0,26	1^a^	0,26	23	5,32
5	7,4	0,051	2,3	0,24	1^a^	0,26	27	5,60
6	7,6	0,049	2,5	0,24	2^b^	0,26	27	5,62
7	7,6	0,051	2,3	0,26	2^b^	0,24	27	5,37
8	7,4	0,049	2,3	0,24	2^b^	0,24	23	5,23

^a^PorosG20 series number 18095 ^ b^Poros G20 series number 18096.

**Table 3:** Parallelism data between calibration curves prepared in mobile phase A with calibration curves prepared in CM PFCHO and CM PFHMII.

Parameters	MPA	CM PFCHO	MPA	CM PFHMII
Correlation coefficient	0.998	0.998	0.998	0.996
Slope	43109	45286	43109	44285
Intercept	−40643	−56821	−40643	−35298
*P* (slopes)	0.000	0.000	0.000	0.000

*P* (between slopes)	0.155		0.486	
*P* (between intercepts)	0.143		0.628	

**Table 4:** Evaluation of accuracy study.

	Mass (*μ*g)				
	2	4	6	8	10
% Recovery elution peak	93.07 ± 1.68	96.04 ± 1.18	97.02 ± 1.71	99.83 ± 1.24	99.51 ± 0.70
